# Wekemo Bioincloud 2026: An AI‐enabled platform for standardized multi‐omics data analyses

**DOI:** 10.1002/imt2.70149

**Published:** 2026-07-08

**Authors:** Yunyun Gao, Guoxing Zhang, Chengrui Wang, Hao Xu, Zhanhui Yu, Shunyao Jiang, Yong‐Xin Liu

**Affiliations:** ^1^ State Key Laboratory of Tropical Crop Breeding, Genome Analysis Laboratory of the Ministry of Agriculture and Rural Affairs, Agricultural Genomics Institute at Shenzhen Chinese Academy of Agricultural Sciences Shenzhen China; ^2^ Digital Intelligence Department Shenzhen Wekemo Technology Group Co. Ltd. Shenzhen China; ^3^ School of Ecology and Nature Conservation Beijing Forestry University Beijing China; ^4^ School of Medicine and Health Harbin Institute of Technology Harbin China; ^5^ Zhengzhou Advanced Research Institute, Harbin Institute of Technology Zhengzhou China; ^6^ Institute of Genomics and Precision Medicine, School of Medical Technology Gannan Medical University Ganzhou China

**Keywords:** artificial intelligence, bioinformatics platform, microbiome, multi‐omics, Wekemo Bioincloud

## Abstract

The rapid expansion of multi‐omics data has created an increasing demand for accessible and reproducible platforms for integrative analysis. Here, we present Wekemo Bioincloud 2026, a comprehensive upgrade of the original platform, providing 41 standardized workflows (expanded from 22) across nine analytical categories, including a newly introduced correlation analysis module for cross‐omics association studies. Visualization capabilities have been substantially expanded to 126 tools (from 65), incorporating artificial intelligence (AI)‐guided parameter selection and automated image interpretation to enhance data exploration and result interpretability. In addition, Wekemo Bioincloud 2026 integrates an AI‐powered research platform that supports the full research lifecycle, including automated literature synthesis, hypothesis generation, intelligent question answering, experimental design, and journal recommendation. Together, these advances reduce technical barriers, improve reproducibility and interpretability, and enable end‐to‐end AI‐assisted multi‐omics research. The platform is freely accessible at https://www.bioincloud.tech/ and supports a broad range of biological and biomedical applications.

## Highlights

- Provides 41 standardized workflows across nine analytical domains, including a new correlation analysis module for cross‐omics integration.
- Expands visualization resources to 126 interactive tools with AI‐guided preprocessing and automated image interpretation.
- Introduces an AI‐powered research platform supporting literature review, hypothesis generation, experimental design, and journal recommendation.

## Graphical abstract

An AI‐powered multi‐omics cloud platform integrating 41 standardized workflows, 126 visualization tools, and full‐lifecycle research intelligence to enable accessible, reproducible, and end‐to‐end integrative multi‐omics analysis.

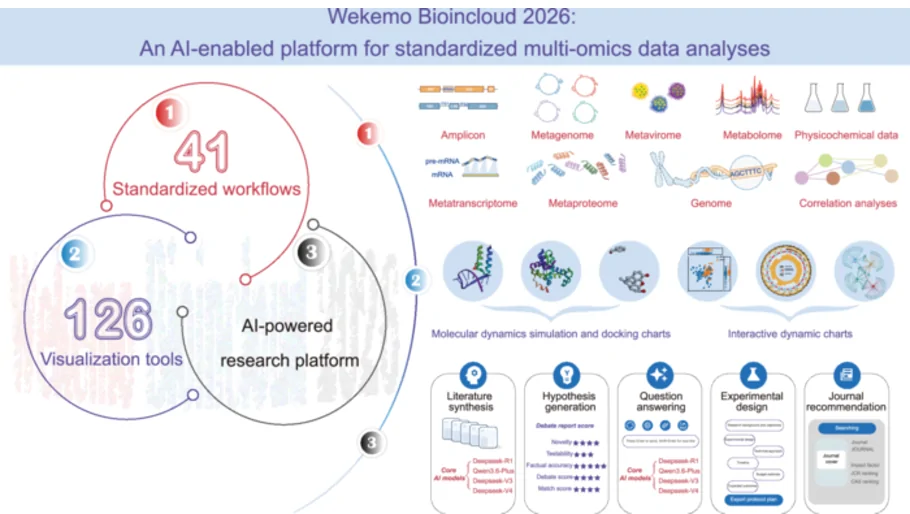

## INTRODUCTION

The past decade has witnessed an explosive growth of multi‐omics data, driven by rapid advances in high‐throughput sequencing, mass spectrometry‐based proteomics and metabolomics, and single‐cell technologies [1, 2, 3]. Genomics, transcriptomics, proteomics, metabolomics, and microbiome profiling are now routinely integrated to characterize biological systems across multiple molecular layers, enabling more comprehensive insights into complex traits, disease mechanisms, and ecosystem functions [4, 5]. This trend has been further accelerated by large‐scale international initiatives and steadily decreasing experimental costs [6, 7], resulting in the accumulation of increasingly heterogeneous omics datasets across diverse biological and environmental contexts.

Despite these opportunities, the analysis and interpretation of multi‐omics data remain nontrivial [3, 8, 9, 10]. The analytical process typically involves multiple sequential steps, including preprocessing, statistical modeling, functional annotation, and cross‐omics integration, which are often implemented using heterogeneous tools with inconsistent input formats and parameter requirements [8, 11, 12]. As a result, users frequently need to manually connect different software components to construct complete analytical pipelines, which increases technical barriers and may compromise reproducibility. In addition, the high dimensionality and intrinsic heterogeneity of multi‐omics datasets pose substantial challenges for visualization and interpretation, particularly in cross‐omics association analyses where multi‐source data must be jointly explored [13, 14]. These limitations collectively hinder efficient data exploration and downstream biological interpretation.

Recent years have seen the rapid emergence of diverse computational ecosystems for multi‐omics analysis, ranging from command‐line frameworks to graphical user interface (GUI)‐based and web‐accessible platforms [15, 16, 17, 18]. These tools have substantially improved the accessibility of omics data analysis and contributed to methodological standardization across different research domains. For example, workflow systems such as Galaxy enable reproducible pipeline construction through modular components, while toolkits such as mixOmics provide robust statistical frameworks for multivariate integration. In addition, platforms such as MetaboAnalyst and similar web‐based systems have simplified metabolomics and multi‐omics statistical analysis for non‐programming users. Collectively, these developments have played an important role in lowering the technical barriers to omics data analysis and promoting reproducibility in computational biology [11]. However, most existing platforms remain largely focused on specific omics layers or isolated analytical stages, and end‐to‐end integration across the full analytical workflow is still limited.

In parallel, recent advances in artificial intelligence (AI), particularly large language models (LLMs) and deep learning‐based approaches, have begun to reshape biomedical data analysis by enabling more flexible data interpretation, automated knowledge extraction, and context‐aware reasoning from complex biological datasets [19, 20, 21]. Beyond traditional machine learning applications in classification and feature selection, emerging studies have demonstrated the potential of AI for literature mining, omics result summarization, and hypothesis generation [20, 21, 22]. In multi‐omics contexts, AI may offer new opportunities to bridge computational outputs and biological interpretation by integrating prior knowledge with data‐driven inference [8]. However, despite these promising developments, the incorporation of AI into multi‐omics platforms remains relatively limited. Most existing systems still rely primarily on predefined statistical pipelines, with minimal support for adaptive reasoning, interactive interpretation, or end‐to‐end scientific assistance [16]. This gap highlights the need for more integrated frameworks that combine standardized multi‐omics analysis with AI‐assisted interpretation and research support.

To address these challenges, we present a systematic upgrade of the first‐generation Wekemo Bioincloud platform [23], resulting in Wekemo Bioincloud 2026. This updated ecosystem optimizes standardized analytical workflows and substantially expands visualization diversity to improve usability and interpretability. Notably, an AI‐powered research platform module is newly introduced to complement data analysis with AI‐assisted research support. Together, these enhancements aim to support the full pre‐ and post‐analysis research workflow, facilitating reproducible, integrative, and AI‐assisted multi‐omics research. The upgraded platform is openly accessible at https://www.bioincloud.tech/.

## RESULTS

### Overview of Wekemo Bioincloud 2026

Compared with the first release, Wekemo Bioincloud 2026 consists of three modules: workflows, visualization tools, and an AI‐powered research platform. Across these modules, the platform now provides 41 standardized workflows (19 newly developed) organized into nine analytical categories. Visualization capabilities have been substantially expanded from 65 to 126 tools. Representative demo datasets and built‐in result interpretation modules are provided within the workflow and visualization modules to facilitate biological interpretation of multi‐omics outputs. Furthermore, Wekemo Bioincloud 2026 integrates an AI‐powered research platform that supports the entire research lifecycle, from hypothesis generation and experimental design to biological interpretation.

To further enhance usability and analytical continuity, Wekemo Bioincloud 2026 adopts a more integrated system‐level design across modules. The platform emphasizes consistent analytical logic through standardized parameter configurations, while preserving flexibility for dataset‐specific customization. In addition, improved cross‐module interoperability enables seamless transitions from data analysis to visualization and interpretation within a unified environment. Collectively, these design improvements reduce workflow fragmentation and support a more coherent and streamlined multi‐omics analysis experience.

### Comprehensive expansion of standardized analysis workflows

**Figure 1:**
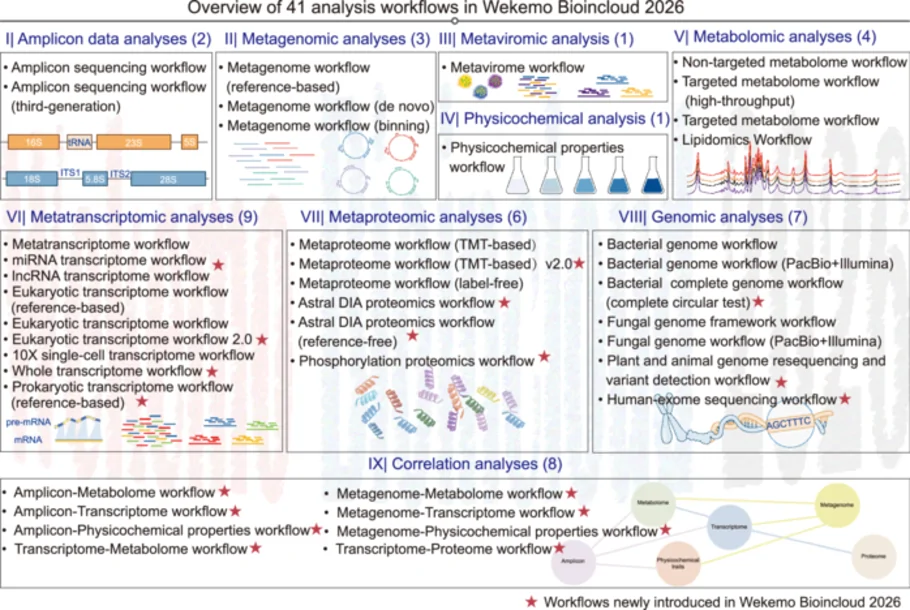
Overview of the 41 analysis workflows in Wekemo Bioincloud 2026. The platform now provides 41 standardized analysis workflows, organized into nine analytical categories, including amplicon, metagenome, metatranscriptome, metaproteome, metavirome, metabolome, genome, physicochemical data, and correlation analysis. Newly introduced workflows in this release are marked with red asterisks.

The workflows module has expanded from 22 to 41 standardized pipelines, organized into nine major analytical categories, including amplicon, metagenome, metatranscriptome, metaproteome, metavirome, metabolome, genome, physicochemical data, and a newly introduced category for correlation analysis (Figure 1). This expansion retains the original eight‐category framework while enabling integrative multi‐omics association analyses. Each workflow includes a demo report, example datasets, step‐by‐step analysis tutorials, and result interpretation guidance.

The 19 newly added workflows are primarily distributed across the metatranscriptomic, metaproteomic, genomic, and correlation analysis domains. In metatranscriptomics, three new pipelines were introduced: a long non‐coding RNA (lncRNA) transcriptome workflow, a eukaryotic transcriptome workflow 2.0, and a whole transcriptome workflow, extending transcript‐level characterization beyond protein‐coding genes. For metaproteomics, additional pipelines were incorporated to support data‐independent acquisition and post‐translational modification profiling, including the TMT‐based metaproteome workflow v2.0, Astral DIA proteomics workflow, reference‐free Astral DIA workflow, and phosphorylation proteomics workflow. In genomics, new workflows were developed to accommodate broader application scenarios, including a bacterial complete genome workflow, a plant and animal genome resequencing and variant detection workflow for population‐level variation analysis, and a human exome sequencing workflow supporting variant identification and annotation from targeted exome data.

A ninth domain, correlation analysis, was introduced to enable integrative comparisons across multiple data types. This domain currently includes cross‐omics association workflows covering amplicon‐metabolome, amplicon‐transcriptome, amplicon‐physicochemical, metagenome‐metabolome, metagenome‐transcriptome, metagenome‐physicochemical, transcriptome‐metabolome, and transcriptome‐proteome analyses. These workflows support pairwise association analyses using standardized statistical and visualization procedures, facilitating exploration of coordinated biological patterns across heterogeneous datasets.

### Enhanced visualization tools with AI‐driven graphical functions

The visualization tools module provides a comprehensive suite of analytical and visualization functions spanning statistical analysis, multi‐omics integration, correlation and pathway analysis, enrichment analysis, evolutionary analysis, and data preprocessing. The total number of visualization tools has increased from 65 to 126. Newly developed tools incorporate guided prompts for data preprocessing and key parameter settings during figure generation, and each tool is accompanied by a step‐by‐step video tutorial. An AI‐based image interpretation function has also been introduced, enabling automatic generation of summaries of key data features to support result interpretation.

**Figure 2:**
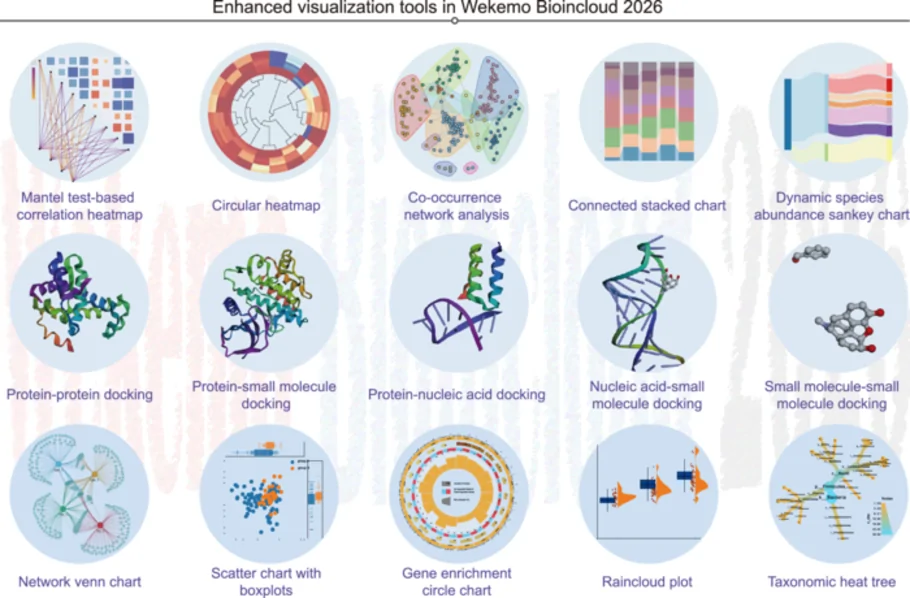
Enhanced visualization tools in Wekemo Bioincloud 2026. The visualization module comprises 126 tools. Shown are representative outputs illustrating major functional expansions, including molecular dynamics simulations, docking analysis, and interactive dynamic charts.

Among the 61 newly added tools (Figure 2), a major expansion focuses on molecular dynamics simulation and docking analyses, including DiffDock‐based docking, protein‐small molecule docking, protein‐protein docking, protein‐peptide docking, small molecule‐small molecule docking, nucleic acid‐small molecule docking, and protein‐nucleic acid docking. The platform also provides interactive dynamic charts, such as species abundance sankey diagrams, circular heatmaps, stacked bar charts, multi‐group scatter plots, kernel density estimation marginal plots, and scatter‐box plots.

Usage statistics indicate that grouped cluster heatmaps, LEfSe plots, and grouped percentage stacked bar plots remain the most frequently used tools. As of January 27, 2026, cumulative usage of the cluster heatmap tool increased from 36,098 to 74,295, LEfSe plots from 34,261 to 89,022, and grouped percentage stacked bar plots from 33,341 to 70,207 since the first release on December 10, 2023. Other highly utilized tools include correlation network analysis (66,449), correlation heatmaps (66,103), redundancy analysis (RDA) and canonical correspondence analysis (CCA) (35,072), Bray–Curtis PCoA analysis (34,108), and principal component analysis (PCA) (30,496).

### An AI‐powered research platform for end‐to‐end scientific support

**Figure 3:**
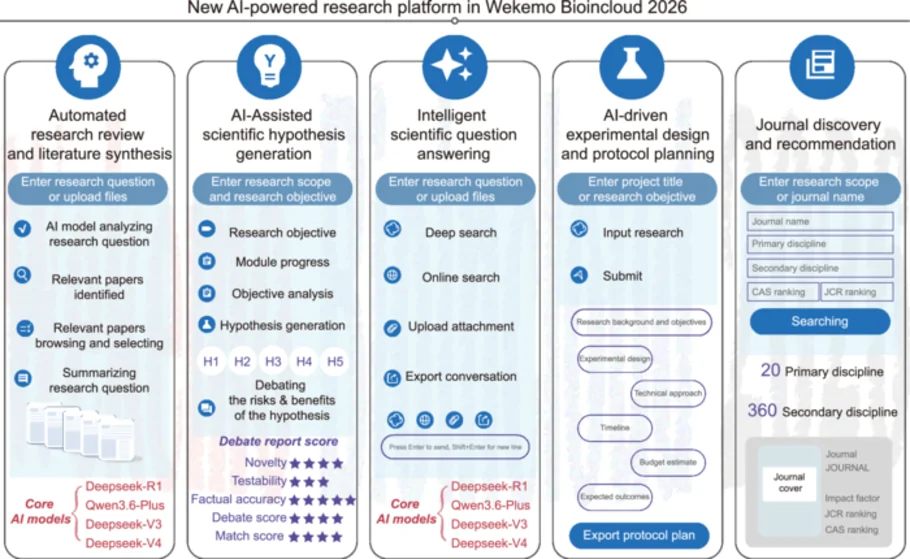
New AI‐powered research platform in Wekemo Bioincloud 2026. Architecture of the AI‐powered research platform module, comprising five functional domains, including automated research review and literature synthesis, AI‐assisted hypothesis generation, intelligent scientific question answering, AI‐driven experimental design and protocol planning, and journal discovery and recommendation. AI, artificial intelligence.

Wekemo Bioincloud 2026 introduces an AI‐powered research platform module designed to accelerate multi‐omics research. A dedicated multi‐omics knowledge base was constructed to support five functional domains: automated research review and literature synthesis, AI‐assisted hypothesis generation, intelligent scientific question answering, AI‐driven experimental design and protocol planning, and journal discovery and recommendation (Figure 3). Together, these domains extend data analysis into a full pre‐ and post‐analysis research chain, enabling end‐to‐end support from scientific review and hypothesis formulation to experimental design and journal selection.

The automated research review domain is powered by multiple AI models (DeepSeek‐R1, DeepSeek‐V3, DeepSeek‐V4, and Qwen3.6‐Plus). After users input a research question or upload files, the system identifies relevant publications and generates structured literature summaries. The hypothesis generation domain produces three to five candidate hypotheses and evaluates them using an internal debate agent across multiple dimensions, including novelty, testability, factual accuracy, and relevance. The intelligent question‐answering domain enables iterative dialogue with deep reasoning and web‐enabled search, continuously refining responses based on user feedback. The experimental design domain generates experimental workflows, technical approaches, timelines, budget estimates, and expected outcomes. The journal recommendation domain leverages a curated knowledge base of 20 primary and 360 secondary disciplines to provide structured journal information and topic‐matched recommendations.

### AI‐assisted interpretation of analytical results

In addition to the AI‐powered research platform, Wekemo Bioincloud 2026 also incorporates AI‐assisted interpretation functions within the workflow and visualization modules. The system can automatically summarize analytical outputs and extract biologically relevant patterns from generated figures, including taxonomic composition analysis, functional pathway analysis, correlation analysis, enrichment analysis, and multi‐omics association results.

**Figure 4:**
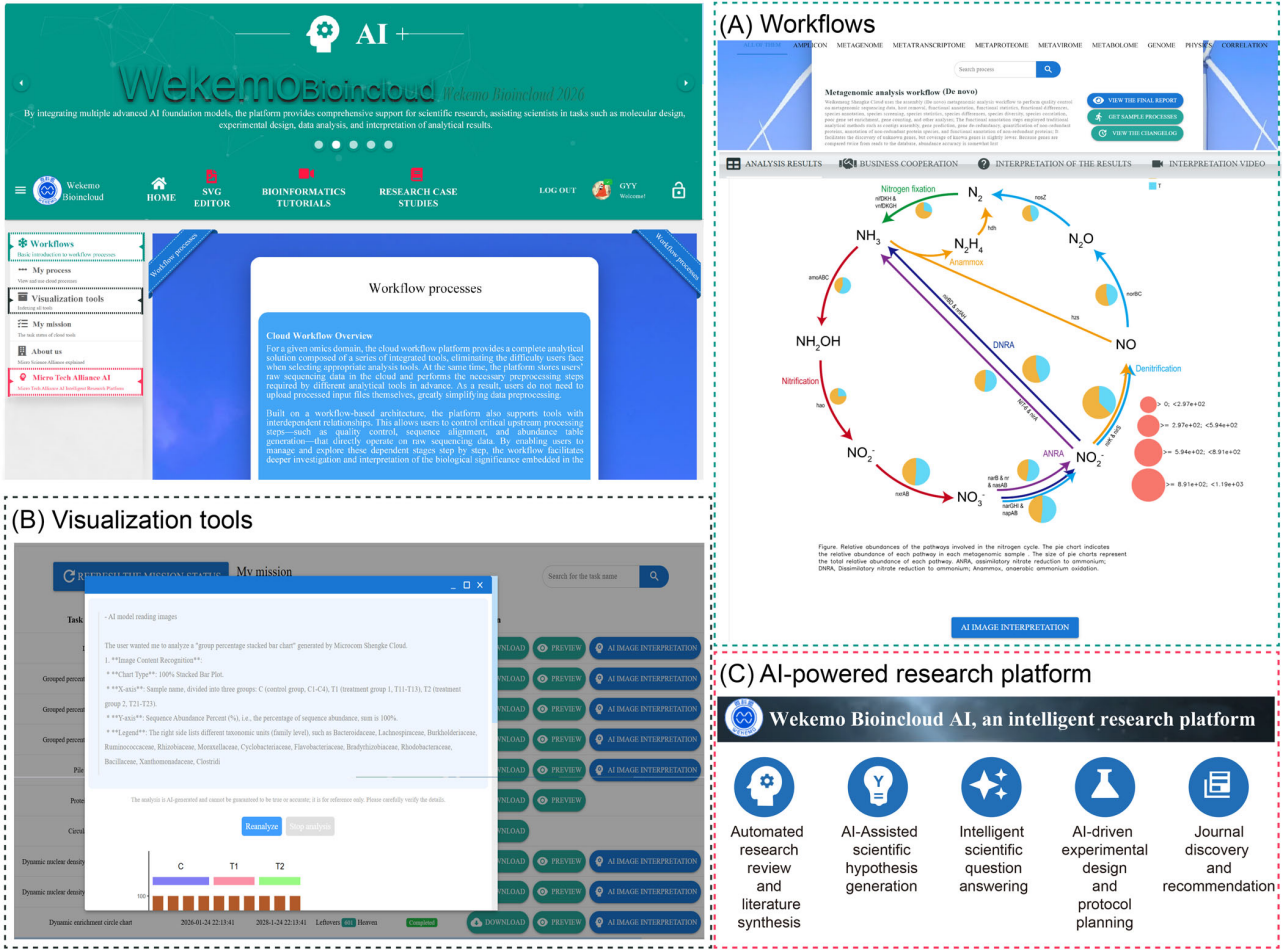
AI‐assisted interpretation and research support functions in Wekemo Bioincloud 2026. Representative examples illustrating AI‐assisted interpretation across different components of the platform. (A) AI‐supported interpretation of workflow analysis results, exemplified by functional interpretation of metagenomic nitrogen cycling pathways. (B) AI‐assisted interpretation of visualization outputs, illustrated by automated analysis of a grouped percentage stacked bar chart. (C) Overview of the AI‐powered research platform, providing intelligent research support through literature synthesis, hypothesis generation, scientific question answering, experimental design, and journal recommendation. AI, artificial intelligence.

For example, the platform supports AI‐assisted interpretation of grouped percentage stacked bar plots by identifying dominant taxa, summarizing community composition patterns, comparing group‐specific differences, and generating biologically meaningful interpretations. Similarly, for elemental cycling analyses (e.g., nitrogen cycling), the system can automatically recognize key functional pathways and genes, evaluate differences among sample groups, infer potential ecological or biological implications, and provide structured summaries together with example scientific text (Figure 4). These functions extend AI applications beyond literature assistance and experimental design, enabling direct interpretation of analytical results and visualization outputs. By integrating figure understanding, biological inference, and scientific writing assistance, the platform helps improve interpretation efficiency and facilitates downstream reporting and knowledge generation.

## DISCUSSION

Compared with the initial release of Wekemo Bioincloud [23], the 2026 version substantially expands both functional scope and analytical strategy. The number of standardized workflows has increased from 22 to 41 across nine analytical domains, including a new correlation analysis category for cross‐omics association studies (e.g., amplicon‐metabolome, metagenome‐transcriptome, transcriptome‐proteome). Together with expanded visualization tools and AI‐assisted parameter guidance, these standardized pipelines reduce technical complexity, improve analytical consistency, and enhance result interpretability. In addition, an AI‐powered research module extends support beyond data analysis to literature synthesis, hypothesis generation, experimental design, and journal recommendation, enabling a more integrated multi‐omics research workflow.

To place Wekemo Bioincloud 2026 within the current landscape, we compared it with several representative multi‐omics and bioinformatics platforms (Supporting Information S1: Table S1). Platforms such as OmicsAnalyst [24] provide powerful visual analytics and causal inference, MetaboAnalyst [17] offers metabolomics‐focused analysis, Galaxy [15] enables flexible and reproducible workflow construction through a modular architecture, and mixOmics [16] delivers advanced multivariate methods for multi‐omics data integration. Transcriptomics‐oriented platforms, including eVITTA [25] and ExpressVis [26], facilitate RNA‐seq analysis and gene expression visualization, whereas ExpOmics [27] supports expression data exploration and cross‐study integration through a web‐based framework. Majorbio Cloud [28] provides standardized microbiome and multi‐omics analysis pipelines with user‐friendly cloud‐based services. While these resources have made valuable contributions, most primarily focus on specific omics layers or analytical stages of the research process and generally provide limited support for AI‐assisted scientific research activities.

In contrast, Wekemo Bioincloud 2026 integrates standardized multi‐omics workflows, comprehensive visualization tools, and an AI‐assisted research platform within a unified, fully web‐based and code‐free environment. In addition to reproducible cross‐omics association analyses, it extends support beyond data analysis to include literature synthesis, hypothesis generation, experimental design, and journal recommendation, thereby bridging the gap between data processing and biological interpretation. A systematic comparison of representative multi‐omics platforms is provided in Supporting Information S1: Table S1, highlighting the functional positioning and unique features of Wekemo Bioincloud 2026.

## CONCLUSION

Wekemo Bioincloud 2026 represents a comprehensive upgrade of the original platform, integrating expanded standardized workflows, enriched visualization capabilities, and an AI‐powered research ecosystem within a unified, web‐based environment. By lowering technical barriers, improving reproducibility, and extending support across the full research lifecycle, the platform enables efficient, integrative, and AI‐assisted multi‐omics studies. As multi‐omics research continues to grow in scale and complexity, Wekemo Bioincloud 2026 provides a flexible and future‐oriented solution that bridges data analysis, interpretation, and scientific discovery. Future development will include a dedicated English‐language interface to improve accessibility and facilitate broader international adoption of the platform. Through continued expansion of analytical and AI‐assisted capabilities, Wekemo Bioincloud 2026 aims to provide accessible and reproducible resources for academic research, supporting a broad community of researchers across biological and biomedical disciplines.

## METHODS

### Platform architecture

Wekemo Bioincloud 2026 is implemented as a web‐based multi‐omics analysis platform with a modular architecture. The front end is developed using JavaScript, HTML, Vue.js, and Bootstrap, enabling responsive interaction and dynamic visualization. Backend services support data preprocessing, workflow execution, and result management, allowing scalable and reproducible analyses across diverse omics datasets.

### Standardized multi‐omics workflows

The platform integrates widely used and community‐validated bioinformatics tools to construct standardized analytical workflows for multi‐omics data analysis. Depending on the workflow, analytical steps may include quality control [23, 29], host contamination removal [30], redundancy reduction [31], binning or reference‐based mapping [32], taxonomic or functional annotation [33, 34, 35, 36, 37], and quantitative profiling [38] of species, genes, metabolites, or proteins. Downstream analyses include diversity estimation, differential analysis, correlation analysis, phylogenetic inference, and functional prediction [39, 40, 41]. These modules are implemented using established statistical and computational methods, each following its corresponding validated analytical framework and reporting statistical measures such as adjusted *p* values/*q* values and effect size estimates where applicable.

Rather than a single fixed pipeline, Wekemo Bioincloud is designed as a flexible analysis platform that allows users to select and configure analytical methods according to experimental design and data characteristics. Accordingly, statistical settings such as filtering criteria, normalization strategies, model selection, and multiple testing correction methods may vary across workflows and user‐defined analyses. Supporting Information S1: Table S2 provides an overview of the implemented analytical modules and associated software tools. Detailed descriptions of workflow options, parameter settings, default configurations, statistical procedures, and software versions are provided in the platform documentation (https://www.bioincloud.tech/), supporting reproducibility and transparency.

To illustrate the workflow design, the amplicon sequencing workflow is provided as a representative example. Amplicon test data were sequenced on the FASTASeq 300 platform at GeneMind [42]. This workflow consists of sequential modules including data preprocessing, ASV generation, taxonomic annotation, basic statistical analysis, diversity analysis, correlation analysis, functional prediction, and visualization. In the data preprocessing stage, raw FASTQ files are processed using user‐defined parameters such as primer sequences, read length thresholds, quality trimming scores (default *Q* = 20), and chimera detection strategies. Denoising and feature inference are performed using the DADA2 pipeline implemented in QIIME 2, including quality filtering, error correction, read merging, and chimera removal, resulting in amplicon sequence variants (ASVs).

For taxonomic annotation, both Naive Bayes classifier (sklearn‐based) and alignment‐based (VSEARCH) methods are supported. Reference databases such as SILVA or Greengenes can be selected, and confidence thresholds (default 0.7 for classifier‐based methods) as well as identity and coverage thresholds (for alignment‐based methods) are adjustable. Downstream analyses include alpha diversity (e.g., Shannon and Simpson indices with optional rarefaction depth setting), beta diversity (e.g., PCoA, NMDS, and PERMANOVA based on Bray–Curtis or UniFrac distances), and differential abundance testing using ANOVA, Kruskal‐Wallis, DESeq 2, or LEfSe depending on data characteristics and study design. Correlation analysis modules support microbial association inference using Pearson, Spearman, or SparCC/propr‐based methods, with user‐defined correlation and significance thresholds. Functional prediction is performed using PICRUSt2 or FAPROTAX to infer putative functional profiles from taxonomic compositions. All analytical outputs are linked to visualization modules, including clustered heatmaps, stacked bar plots, Sankey diagrams, phylogenetic trees, and network graphs.

The platform additionally incorporates an AI‐assisted interpretation module for analytical outputs and visualizations (Figure 4A). This module is applied to summarize statistical results and extract key patterns from generated figures, including taxonomic composition profiles, differential abundance results, correlation structures, and functional prediction outputs. The AI module is designed to provide descriptive summaries of analytical outputs based on input results and does not alter the underlying statistical calculations. It supports interpretation of multiple omics layers and assists users in reviewing and summarizing analytical findings for downstream analysis and reporting.

### Visualization tools

The visualization module integrates analytical plotting and data exploration functions for statistical analysis, multivariate analysis, correlation and network analysis, enrichment analysis, evolutionary analysis, and multi‐omics data visualization [40, 41, 43]. The toolkit covers a broad spectrum of commonly used analytical scenarios, including basic statistical visualization (e.g., clustered heatmaps, grouped percentage stacked bar plots, and Venn or flower plots), ordination‐based multivariate analyses (e.g., Bray–Curtis principal coordinates analysis [PCoA], PCA, nonmetric multidimensional scaling [NMDS], and distance‐based redundancy analysis [dbRDA]), differential abundance and group comparison methods (e.g., LEfSe, orthogonal partial least squares discriminant analysis [OPLS‐DA], and Kruskal–Wallis tests), correlation‐based analyses (e.g., correlation heatmaps, correlation networks, RDA, and CCA), as well as pathway‐level visualizations including KEGG enrichment analysis for untargeted metabolomics and metabolite‐annotated KEGG pathway mapping.

To improve usability and reproducibility, each visualization tool is accompanied by example datasets and step‐by‐step tutorials that guide users through data formatting, preprocessing, and figure generation. Users can customize analytical parameters according to their study design, including group assignment schemes, color schemes, and normalization strategies. For example, in LEfSe analysis, users can adjust normalization scaling factors and LDA score thresholds; in clustered heatmap visualization, options are provided for grouping strategy, feature selection limits, and data transformation methods (e.g., *Z*‐score normalization, log10 transformation, or raw values). For correlation network analysis [44, 45], users may define feature number limits, correlation methods (Spearman, Pearson, or Kendall), correlation thresholds, and statistical significance cutoffs.

In addition, the platform supports AI‐assisted interpretation of generated figures (Figure 4B), enabling automated summarization of key statistical patterns and biologically relevant features. This includes identification of dominant taxa or metabolites, detection of group‐specific differences, and generation of structured interpretation text to facilitate downstream biological interpretation and manuscript preparation.

### AI‐powered research platform

Wekemo Bioincloud 2026 incorporates an AI‐powered research platform built upon a curated multi‐omics knowledge base [20, 21, 22]. Multiple LLMs, including DeepSeek‐R1, DeepSeek‐V3, DeepSeek‐V4, and Qwen3.6‐Plus, are integrated to support automated literature summarization, hypothesis generation, scientific question answering, experimental design, and journal recommendation (Figure 4C). Model selection is user‐configurable rather than automatically assigned by backend routing strategies.

Different AI‐assisted modules mainly differ in the contextual information and analytical workflows provided to the models. Literature‐related tasks primarily analyze publication content, including titles, keywords, abstracts, figures, and associated text. Experimental design additionally incorporates user‐provided research background, available resources, existing results, and methodological information from related studies. The hypothesis generation module further implements a multi‐agent evaluation process to assess candidate hypotheses based on novelty, feasibility, supporting evidence, and biological relevance. The underlying knowledge base is updated daily using curated literature resources and related databases.

AI‐guided parameter recommendation is implemented using expert‐defined analytical rules together with dataset‐derived characteristics, including sample size, variable type, group structure, and distribution patterns, and subsequently recommends appropriate preprocessing methods, statistical models, and visualization parameters, thereby improving analytical consistency and reducing user‐dependent variability.

## AUTHOR CONTRIBUTIONS

**Yunyun Gao:** Conceptualization; validation; formal analysis; investigation; writing—original draft; writing— review and editing; visualization; funding acquisition. **Guoxing Zhang:** Methodology; software; validation; formal analysis; investigation; resources; data curation; writing—review and editing. **Chengrui Wang**: Methodology; software; validation; formal analysis; investigation; resources; writing—review and editing. **Hao Xu**: Methodology; software; validation; writing—review and editing. **Zhanhui Yu**: Methodology; software; validation; writing—review and editing. **Shunyao Jiang**: Conceptualization; methodology; software; validation; formal analysis; investigation; resources; data curation; writing—review and editing; supervision. **Yong‐Xin Liu**: Conceptualization; validation; formal analysis; investigation; resources; writing—original draft; writing—review and editing; supervision; funding acquisition. All authors have read the final manuscript and approved it for publication.

## CONFLICT OF INTEREST STATEMENT

The authors declare no conflicts of interest.

## ETHICS STATEMENT

No animals or humans were involved in this study.

## Supporting information


**Table S1:** Feature comparison of Wekemo Bioincloud 2026 with representative multi‐omics platforms.
**Table S2:** Software and packages used in the corresponding workflows of Figure 1.

## Data Availability

Data sharing not applicable to this article as no datasets were generated or analysed during the current study. No new data or code were generated in this study. All demonstration data for visualization purposes are available on the Wekemo Bioincloud website (https://www.bioincloud.tech/). Supplementary materials (tables, graphical abstract, slides, videos, Chinese translated version and update materials) may be found in the online DOI or iMeta Science http://www.imeta.science/.
